# METTL3 in Cr (VI)-induced carcinogenesis and CXCL6 expression associated with lung cancer development

**DOI:** 10.1016/j.gendis.2025.101778

**Published:** 2025-07-24

**Authors:** Jie Liu, Xiao Han, Fan-Li Sun, Xue Wang, Lin Wang, Yan-Qiu Zhao, Wen-Jing Liu, Bing-Hua Jiang

**Affiliations:** a Department of Oncology, Affiliated Cancer Hospital of Zhengzhou University & Henan Cancer Hospital, Zhengzhou, Henan 450008, China; b Henan International Joint Laboratory of Lung Cancer Biology and Therapeutics, Zhengzhou, Henan 450008, China; c Department of Prenatal Diagnosis Center, The Third Affiliated Hospital of Zhengzhou University, Zhengzhou University, Zhengzhou, Henan 450052, China; d Academy of Medical Science, Zhengzhou University, Zhengzhou, Henan 450000, China

Environmental carcinogens from air pollution and metal exposure have emerged as major sources for inducing cancers. Epidemiological data have shown that hexavalent chromium (Cr [VI]) is linked to cancer development. A list of evidence supports the association between occupational exposure to hexavalent chromium and various cancers, especially lung cancer.^1^ However, the underlying mechanisms by which Cr (VI) induces cancers are still unclear. As the most prevalent mRNA modification, N^6^-methyladenosine (m6A) provided a novel form of post-transcriptional gene regulation. m6A regulators were found to be closely correlated with specific malignant tumors, including lung cancer. Among these, METTL3, the core methyltransferase for m6A modification, has gradually attracted much attention due to its cancer-promoting role in multiple cancers.^2^ However, the exact roles of METTL3 and m6A methylation in carcinogenesis induced by chronic chromium exposure and lung cancer development remain unclear.

To explore the functional effects of METTL3 and m6A methylation on Cr (VI)-induced carcinogenesis and lung cancer development, METTL3 mRNA expression levels were investigated between tumor tissues and adjacent normal tissues across 22 TCGA tumor types via TIMER, a web tool for variant analysis of The Cancer Genome Atlas (TCGA) data. As shown in Figure 1A, METTL3 levels were markedly elevated across 14 tumor types, including lung adenocarcinoma (LUAD) and lung squamous cell carcinoma (LUSC). Our team successfully established Cr (VI)-transformed (Cr-T) cells by transforming non-tumorigenic human lung epithelial BEAS-2B cells through long-term exposure to Cr (VI).^1^ Cr-T cells showed typical malignant transformation and tumorigenic properties. Compared to those in parental BEAS-2B cells, METTL3 expression levels and total m6A levels in Cr-T cells were significantly higher in the transformed cells. We knocked down METTL3 expression in Cr-T cells (METTL3-KD) using SMARTpool-human METTL3 siRNAs (L-005170-02-0005, Dharmacon) and found that METTL3 silencing decreased the m6A content (Fig. 1B). Also, higher Cr (VI) concentrations contributed to the increase of overall m6A levels in BEAS-2B cells (Fig. 1C). DAA is an S-adenosylmethionine synthesis inhibitor that inhibits the internal m6A methylation level. The m6A content levels were decreased in response to DAA treatment (Fig. 1C). Stable METTL3 knockout (METTL3-KO) Cr-T cells were established using a CRISPR/Cas9-edited system (Synthego Corporation, Redwood City, CA, USA). Total m6A levels were significantly reduced after METTL3 was knocked out in the cells (Fig. 1D). METTL3 KO/KD cells had significantly lower rates of wound healing (Fig. 1E; Fig. S1A), proliferative rates (Fig. 1F; Fig. S1B), migrational capacity (Fig. 1G; Fig. S1C), and clonogenic abilities (Fig. 1H; Fig. S1D) than the control cells. Cr-T cells were then transfected with the pcDNA3/Flag-METTL3 plasmid (Addgene; plasmid 53739), and an empty pcDNA3 vector was used as the control. Western blotting and qRT-PCR analysis were used to test the efficacy of METTL3 overexpression (Fig. 1I and J). As we predicted, the forced expression of METTL3 significantly increased the rates of wound healing (Fig. S1E), migrational rates (Fig. S1G), proliferative capacity (Fig. S1F), and clonogenic abilities (Fig. S1H) compared to those of the control cells. We also found that human lung cancer cell lines and Cr-T cells dramatically increased METTL3 levels (Fig. 1K). We then knocked down METTL3 expression in the A549 and H2030 cell lines, and the knockdown efficacy of METTL3 was confirmed by Western blotting (Fig. 1L). Similar functional experiments were carried out in two different types of cell lines. The knockdown of METTL3 also reduced the cell migration and proliferation in lung cancer cell lines (Fig. S1I‒S1L).

**Figure 1:**
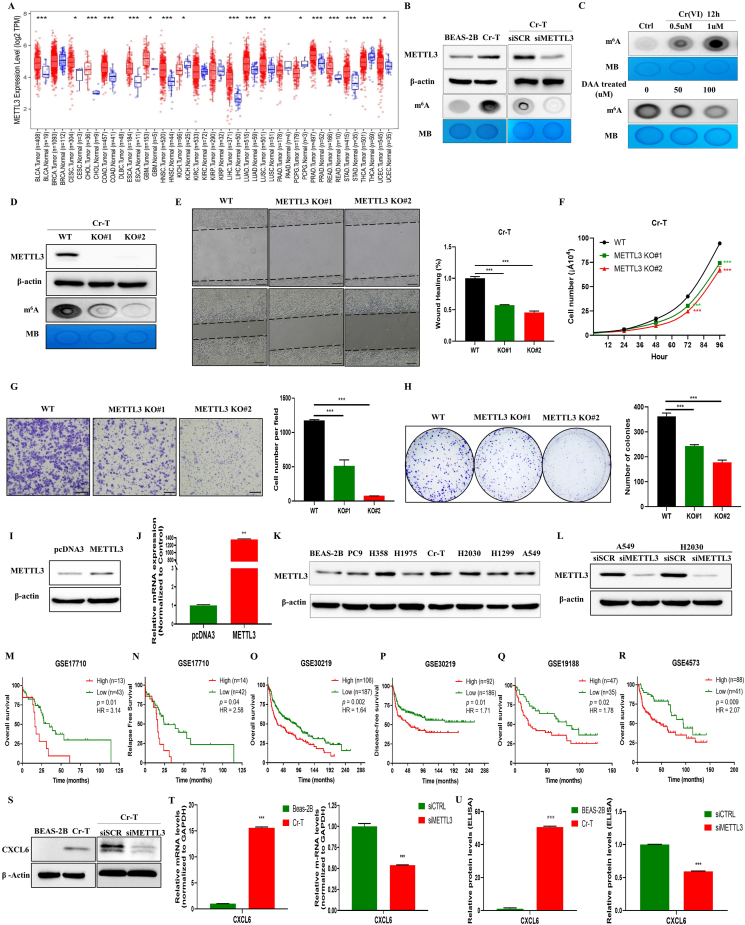
The expression and role of METTL3 in Cr (VI)-induced carcinogenesis and cancer development. **(A)** METTL3 mRNA expression levels in lung tumor tissues and adjacent normal tissues across 22 different types of cancer tissues. **(B)** Western blotting analysis of METTL3, β-actin, m6A levels, and MB in BEAS-2B, Cr-T, and the cells expressing non-targeting control or METTL3 siRNAs (siSCR or siMETTL3): Cr-T cells were transfected with ON-TARGETplus SMART pool siRNAs targeting METTL3 or with a non-targeting control (50 nM) for 48 h. **(C)** BEAS-2B cells were treated with 0.5 μM and 1 μM Cr (VI), and m6A levels were analyzed. Cr-T cells were treated with DAA at concentrations of 0, 50, and 100 μM for 48 h. The m6A content levels in the cells were analyzed. **(D)** Western blotting and m6A dot blot analysis indicated the METTL3 and the m6A content levels were greatly decreased in Cr-T cells with the METTL3 knockout. **(E**–**H)** The results of the wound-healing assay (E), cell proliferation assay (F), Transwell migration assay (G), and colony formation assay (H) were analyzed in the Cr-T cells with or without METTL3 knockout. Scale bar refers to 100 μm. **(I**–**J)** Western blotting (I) and qRT-PCR (J) analysis were used to verify the efficacy of METTL3 overexpression. **(K)** Western blotting analysis of METTL3 expression levels in multiple lung cancer cell lines and Cr-T cells. **(L)** Western blotting analysis of METTL3 levels in A549 and H2030 cells transfected with non-targeting control or METTL3 siRNAs (siSCR or siMETTL3): A549 and H2030 cells were transfected with ON-TARGETplus SMART pool siRNAs targeting METTL3 or with a non-targeting control (50 nM) for 48 h. **(M**–**R)** Overall survival curves of the patients with high (red) and low (green) METTL3 expression levels were plotted using GraphPad Prism in the GSE17710, GSE30219, GSE4573, and GSE19188 datasets. **(S)** Western blot analysis showed CXCL6 levels in BEAS-2B and Cr-T cells with or without METTL3 silencing. **(T, U**) METTL3 knockdown decreased CXCL6 expression levels in Cr-T cells at the RNA level via real-time PCR (T) and at the secreted protein level via ELISA assay (U). TCGA, The Cancer Genome Atlas. MB, methylene blue. DAA, 3-deazaadenosine. WT, wild type. KO, knockout. Data were presented as the X ± SEM (*n* = 3). HR, hazard ratio. DFS, disease-free survival. OS, overall survival. ∗ indicates a significant difference at *P* < 0.05; ∗∗ at *P <* 0.01; ∗∗∗ at *P* < 0.001.

We then analyzed the protein levels of METTL3 in pan-cancer via the UALCAN database and showed that METTL3 protein expression levels were up-regulated in different types of cancers, including breast cancer, ovarian cancer, and LUAD (Fig. S1M). We also found that changes of ten m6A RNA methylation regulators, including the up-regulation of METTL3, showed distinct genetic alterations in LUAD and LUSC tissues using data from the TCGA dataset (PanCancer Altas). Among the ten m6A-associated genes, METTL3 was amplified more frequently in LUAD and LUSC (Fig. S2A). Besides, the copy number of METTL3 showed positive correction with its mRNA expression level in both LUAD and LUSC tissues (Fig. S2B). An additional study indicated a significant up-regulation of METTL3 gene expression in lung tumors compared to normal tissues, as evidenced by analysis of four distinct Gene Expression Omnibus (GEO) datasets, namely, GSE2514, GSE7670, GSE40791, and GSE19804 (Fig. S2C). We then carried out Kaplan–Meier survival analysis to explore the correlation of METTL3 overexpression with the prognosis of human lung cancer patients by analyzing cohorts of four independent Gene Expression Omnibus (GEO) datasets (GSE17710, GSE30219, GSE4573, and GSE19188). The results suggested that higher METTL3 expression levels exhibited a poorer prognosis (Fig. 1M–R). These results highlight the importance of higher METTL3 expression levels as a prognostic marker in lung cancer.

We then investigated the co-expressed genes of METTL3 in 522 LUAD cases and 504 LUSC cases through the LinkedOmics database. Biological process (BP) pathway analysis of METTL3 molecular function were carried out via GSEA. The BP results in LUAD showed that METTL3-associated differentially expressed genes (DEGs) were primarily enriched in RNA modification pathways, RNA splicing, mitochondrial gene expression, leukocyte cell–cell adhesion, and the integrin-mediated signaling pathway (Fig. S2D). The BP results in LUSC showed that METTL3-associated DEGs were primarily enriched in molecules that regulate RNA modifications, mRNA processing, the meiotic cell cycle, immune effector process regulation, and cell-substrate adhesion (Fig. S2E). Both analyses in LUAD and LUSC indicated that METTL3 plays an important role in global post-transcriptional modifications of mRNAs, immune response regulation, and focal adhesion. Western blot analysis further revealed significantly higher CXCL6 expression levels in Cr-T cells than in parental B2B cells, whereas METTL3 knockdown greatly decreased CXCL6 levels in these cells (Fig. 1S). These results were further validated by a separate experiment at the RNA level via real-time PCR (Fig. 1T) and at the secreted protein level via ELISA assays (Fig. 1U). Meanwhile, we also showed that higher expression levels of CXCL6 are associated with different types of human cancers (Fig. S2F) and that CXCL6 protein levels are markedly higher in LUSC analyzed by using the UALCAN database (Fig. S2G).

The biological functions of m6A regulators and m6A modifications in carcinogenesis and tumor development have been widely studied recently but often exhibit complexity and diversity. Writers (METTL3, METTL14, and WTAP) and erasers (FTO and ALKBH5) theoretically seem to play opposing roles in biological functions. m6A modification has complex and diverse effects on carcinogenesis and tumor development. However, the underlying mechanisms remain to be elucidated. One of the reasons is inter-tumor and intra-tumor heterogeneity. The other reason may be that m6A modifications are global, dynamic, and reversible, and the recognition of different readers may also play important roles in different stages of tumor development. In this study, we confirmed that METTL3 expression level was significantly elevated in Cr (VI)-transformed cells and was essential for Cr (VI)-induced carcinogenesis and lung cancer development. Recent studies have suggested that METTL3 functions as an oncogene by mediating the m6A modifications of EZH2 and MYC, which contributes to the development of cancers.^3^ Interestingly, chronic Cr (VI) exposure can induce cancer stem cell-like properties and cell transformation by significantly increasing EZH2 and MYC expression levels.^4^^,^^5^ Therefore, we speculate that the regulation of the m6A modifications of EZH2 and MYC may be one of the reasons that METTL3 promotes Cr (VI)-induced carcinogenesis. However, the precise mechanism by which METTL3 promotes Cr (VI)-induced carcinogenesis is not fully understood. According to the GSEA analysis, METTL3 was primarily enriched in the global post-transcriptional modifications of mRNAs in LUAD and LUSC. These results suggest that METTL3 may act as an oncogene via a complicated post-transcriptional network. In this study, we also demonstrated that METTL3 regulates the expression of CXCL6, which is associated with lung cancer development. Based on the database analysis, we speculate that METTL3 may indirectly regulate the expression of CXCL6. Our new results show that METTL3 increases CXCL6 expression through HIF-1α (Fig. S2H and S2I).

Combined with recent studies, these findings suggest that one of the pro-tumoral activities of METTL3 is through the regulation of immune-related pathways and that METTL3 may be a potential therapeutic target in anticancer immunotherapy. However, the detailed molecular mechanism of METTl3 needs further investigation. Our findings reveal that METTL3 plays an essential role in Cr (VI)-induced carcinogenesis and that the expression of CXCL6 is associated with lung cancer development. Here, our research presents a novel mechanism of Cr (VI) carcinogenicity, and METTL3 may be a potential therapeutic target for lung cancer.

## CRediT authorship contribution statement

**Jie Liu:** Writing – original draft, Methodology, Funding acquisition, Data curation, Conceptualization. **Xiao Han:** Software, Project administration, Formal analysis, Data curation. **Fan-Li Sun:** Software, Resources, Methodology, Investigation. **Xue Wang:** Validation, Software, Methodology, Investigation. **Lin Wang:** Validation, Supervision, Software, Resources. **Yan-Qiu Zhao:** Supervision, Project administration, Methodology. **Wen-Jing Liu:** Visualization, Supervision, Funding acquisition, Conceptualization. **Bing-Hua Jiang:** Writing – review & editing, Validation, Supervision, Methodology, Funding acquisition, Conceptualization.

## Funding

This work was supported by the 10.13039/501100001809National Natural Science Foundation of China (No. 82102916, 82073393) and the Medical Science and Technology Project of Henan Province, China (No. SBGJ202302023).

## Conflict of interests

Bing-Hua Jiang is one of the Associate Editors of Genes & Diseases, and he has no involvement in the peer-review of this article and no access to information regarding its peer-review. Other authors have no competing interests to declare.
