# Supplementary material for: METTL3 in Cr (VI)-induced carcinogenesis and CXCL6 expression associated with lung cancer development
Source: Genes Dis. 2025 Jul 24;13(2):101778. doi: 10.1016/j.gendis.2025.101778 (PMC12682016; doi:10.1016/j.gendis.2025.101778)
Supplement: Multimedia component 1 [file mmc1.docx]

**Supplementary Material**

**Supplementary materials and methods**

**Cell culture**

The human bronchial epithelial BEAS-2B cells and the human lung cancer cell lines (H1975, H358, H1299, A549, H2030, and PC9) were obtained from American Type Culture Collections (Manassas, VA, USA). All the cells except BEAS-2B and Cr-T were cultured in the RPMI 1640 medium (Invitrogen, Carlsbad, CA, USA) supplemented with 10 % fetal bovine serum (FBS) and antibiotics. BEAS-2B cells were cultured in Dulbecco's Modified Eagle's medium (Invitrogen, Carlsbad, CA, USA) supplemented with 10% FBS and antibiotics. BEAS-2B cells were maintained with DMEM medium containing 0.5 μM Cr (VI) for six months to select Cr-resistant cells. Parallel cultures grown in Cr–free DMEM acted as passage-matched controls. After long-term exposure to Cr (VI), Cr-T cells are confirmed to have the abilities to transform and grow into tumors as we previously described ^1^.

**Immunoblotting and antibodies**

The Western Blot assay was conducted as previously described ^1^. Briefly, the cells were washed in cold 1× PBS buffer and lysed in ice-cold RIPA buffer supplemented with protease inhibitors on ice for 30 min. The protein concentrations were quantified by the BCA method according to the manufacturer's instructions. The protein samples were subjected to SDS–polyacrylamide gel electrophoresis (SDS-PAGE) then transferred to membranes (BioRad, USA). The membrane was blocked with 5% non-fat milk for one hour, followed by overnight incubation with primary antibodies diluted in 2% BSA. After washing, the membranes were incubated with HRP-conjugated secondary antibodies for one hour then exposed to an imager (Bio‐rad ChemiDoc MP, Bio‐rad). The following antibodies were used: METTL3 (#96391, Cell Signaling Technology, USA), CXCL6 (#ab9324, Abcam, UK), HIF1α (#565924, BD Biosciences, USA), and β-actin (#ab8227, Abcam, UK). Blots were visualized with the enhanced chemiluminescence reagent Supersignal (Pierce, Rockford, IL, USA).

**RNA extraction and qRT-PCR**

Total RNA extraction and reverse transcriptase‐polymerase chain reaction (qRT-PCR) assays were performed as previously described ^2^. Briefly, Total RNAs were isolated from cells using Trizol (Invitrogen, CA, USA) according to the manufacturer's instructions. The cDNA synthesis was performed using M-MLV reverse transcriptase (Invitrogen) from 1μg of total RNAs. qRT-PCR was conducted using the Power SYBR Green PCR Master Mix kit (Applied Biosystems, Carlsbad, CA, USA) and carried on a LightCycler 480 Real-Time PCR System (Roche Applied Science). The primers used for qRT-PCR were as follows: METTL3, forward 5'-CTATCTCCTGGCACTCGCAAGA-3' and reverse 5'-GCTTGAACCGTGCAACCACATC-3'; CXCL6, forward 5'-TTCGGTCCAGTTGCCTTCTC-3' and reverse 5'- TCTTCTCCTGGGGGTACTGG-3'; GAPDH, forward 5'-GGAGCGAGATCCCTCCAAAAT-3' and reverse 5'-GGCTGTTGTCATACTTCTCATGG-3'. Their expression levels were normalized to glyceraldehyde 3-phosphate dehydrogenase (GAPDH). The Ct (2−ΔΔCt) method was used in the analysis of PCR data. All reactions were performed in triplicate.

### Cell proliferation, colony formation, wound-healing, and Trans-well assay

For the cell proliferation assay, the target cells were seeded in 24-well plates at 1.2 × 10^4^ cells per well. The number of cells was then monitored daily, and data were collected from three separate experiments with four replications each time. Colony formation, wound-healing, and Trans-well assay were conducted as previously described ^3^. Briefly, the colony formation assay was performed using six-well plates. Six hundred target cells were cultured for eight days, and the number of colonies were counted after the fixing and staining. For the wound-healing assay, when the target cells were cultured to 100% confluence, a pipette tip (200 μL) was utilized to scrape the cell layer on the plate. After incubation with a serum-free medium for 24-48 hours, the cellular migration status will be observed. The Transwell migration assay was carried out using the Transwell chamber (#MCEP24H48, Millipore, USA). Target cells (1 × 10^5^) were cultured with 200ul serum-free medium for 18h. After incubation, the cells were fixed in methanol for 15 minutes then stained with 0.1% crystal violet (Sigma-Aldrich, USA) for 30 minutes. The number of migrated cells was photographed and counted after fixing and staining.

**Transfections and establishment of stable knockout cells**

The cells (1× 10^5^) were seeded in six‐well plates and transfected using jetPRIME reagent (Polyplus transfection) with 110 pmol of ON‐TARGETplus SMARTpool‐Human METTL3 siRNA (L-005170-02-0005, Dharmacon) or ON‐TARGETplus Nontargeting Pool (D-001810-10-50, Dharmacon) according to the manufacturer's protocols. ON-TARGETplus SMARTpool was a mix of four different siRNAs, and target sequences of METTL3 were shown in supplementary data 1. CRISPR/Cas9-edited METTL3-knock-out Cr-T cells were designed and purchased from Synthego Corporation. The [pcDNA3/Flag-METTL3](https://www.addgene.org/53739/) plasmid used for the over-expression of Cr-T cells was a kind gift from Dr. Chuan He (Chicago University, USA).

### Quantification of the m6A modification and dot blot assay

For dot blot assay, 500 ng of RNA samples were loaded on an N+ membrane (#RPN303B, GE Health, USA), then UV cross‐linked. Methylene blue (#M9140‐25G, Sigma‐Aldrich, USA) was used to ensure an equal amount of total RNAs. The membrane was blocked with 5% non-fat milk for one hour, followed by overnight incubation with an anti-m6A polyclonal antibody (#202003, Synaptic Systems, Germany). After washing, the membranes were incubated with HRP-conjugated secondary antibodies for one hour and then exposed to an imager (Bio‐rad ChemiDoc MP, Bio‐rad).

**ELISA assay**

CXCL6 expression levels were detected in cell culture supernatants obtained from different groups as indicated. The concentrations were analyzed by using the human CXCL6 Quantikine ELISA kit (#D6050, R&D Systems, UK) according to the manufacturer's instruction.

**Bioinformatic analysis**

The pan-cancer expression profiling of METTL3 was visualized through the TIMER platform ^4^. Genome and RNA sequencing results for lung cancer were downloaded from the cbioportal database ^5^. The correlations between METTL3 and immune cell infiltration in pan-cancer were assessed by the TISIDB database ^6^. The UALCAN database now provides the protein expression analysis using data from the Clinical Proteomic Tumor Analysis Consortium (CPTAC) Confirmatory/Discovery dataset^7^. We then evaluated the protein expression levels of METTL3 in pan-cancer by CPTAC analysis. Co-expression networks and gene set enrichment analysis (GSEA) of METTL3 were performed via the LinkedOmics database ^8^. A total of 8 independent lung cancer datasets (GSE2514 ^9^, GSE7670 ^10^, GSE40791 ^11^, GSE19804 ^12^, GSE17710 ^13^, GSE30219 ^14^, GSE19188 ^15^, and GSE4573 ^16^) from the Gene Expression Omnibus (GEO) database were used and analyzed. GSE2514, GSE7670, GSE40791, and GSE19804, which included normal controls, were studied to verify the METTL3 expression in normal and cancer tissues. GSE17710, GSE30219, GSE19188, and GSE4573 contained survival information and were explored the prognostic value of METTL3. Data from GEO were downloaded by the R package of GEOquery ^17^. For multiple probe set signals of the same gene ID, the maximum value was retained as gene value. As for the prognostic analysis of METTL3 in different data sets, all patients were classified into two groups according to METTL3 expression levels at all possible cutoff points. The log-rank analysis is used to calculate the risk difference between any two groups. Then we analyzed the risk difference between any two groups and selected the cutoff point with the most significant P-value to be displayed in this study.

**Statistical Analysis**

SPSS 19.0 (SPSS Inc, Chicago, IL, USA) and GraphPad Prism 7.0 (GraphPad Software Inc, San Diego, CA, USA) were used for statistical assessment. Kaplan-Meier curve was generated to estimate the prognosis. The data are presented as the mean ± standard error means from independent experiments followed by analysis of Student's t-test or Pearson correlation analysis. A p-value of less than 0.05 was considered as significant.

**Supplementary references**

1. Wang L, Qiu JG, He J, et al. Suppression of miR-143 contributes to overexpression of IL-6, HIF-1alpha and NF-kappaB p65 in Cr(VI)-induced human exposure and tumor growth. Toxicol Appl Pharmacol. 2019;378:114603.

2. He J, Qian X, Carpenter R, et al. Repression of miR-143 mediates Cr (VI)-induced tumor angiogenesis via IGF-IR/IRS1/ERK/IL-8 pathway. Toxicol Sci. 2013;134(1):26-38.

3. Xie H, Li J, Ying Y, et al. METTL3/YTHDF2 m(6) A axis promotes tumorigenesis by degrading SETD7 and KLF4 mRNAs in bladder cancer. J Cell Mol Med. 2020;24(7):4092-4104.

4. Li T, Fu J, Zeng Z, et al. TIMER2.0 for analysis of tumor-infiltrating immune cells. Nucleic Acids Res. 2020;48(W1):W509-W514.

5. Gao J, Aksoy BA, Dogrusoz U, et al. Integrative analysis of complex cancer genomics and clinical profiles using the cBioPortal. Sci Signal. 2013;6(269):pl1.

6. Ru B, Wong CN, Tong Y, et al. TISIDB: an integrated repository portal for tumor-immune system interactions. Bioinformatics. 2019;35(20):4200-4202.

7. Chandrashekar DS, Bashel B, Balasubramanya SAH, et al. UALCAN: A Portal for Facilitating Tumor Subgroup Gene Expression and Survival Analyses. Neoplasia. 2017;19(8):649-658.

8. Vasaikar SV, Straub P, Wang J, Zhang B. LinkedOmics: analyzing multi-omics data within and across 32 cancer types. Nucleic Acids Res. 2018;46(D1):D956-D963.

9. Su LJ, Chang CW, Wu YC, et al. Selection of DDX5 as a novel internal control for Q-RT-PCR from microarray data using a block bootstrap re-sampling scheme. BMC Genomics. 2007;8:140.

10. Stearman RS, Dwyer-Nield L, Zerbe L, et al. Analysis of orthologous gene expression between human pulmonary adenocarcinoma and a carcinogen-induced murine model. Am J Pathol. 2005;167(6):1763-1775.

11. Zhang Y, Foreman O, Wigle DA, et al. USP44 regulates centrosome positioning to prevent aneuploidy and suppress tumorigenesis. J Clin Invest. 2012;122(12):4362-4374.

12. Lu TP, Tsai MH, Lee JM, et al. Identification of a novel biomarker, SEMA5A, for non-small cell lung carcinoma in nonsmoking women. Cancer Epidemiol Biomarkers Prev. 2010;19(10):2590-2597.

13. Wilkerson MD, Yin X, Hoadley KA, et al. Lung squamous cell carcinoma mRNA expression subtypes are reproducible, clinically important, and correspond to normal cell types. Clin Cancer Res. 2010;16(19):4864-4875.

14. Rousseaux S, Debernardi A, Jacquiau B, et al. Ectopic activation of germline and placental genes identifies aggressive metastasis-prone lung cancers. Sci Transl Med. 2013;5(186):186ra166.

15. Hou J, Aerts J, den Hamer B, et al. Gene expression-based classification of non-small cell lung carcinomas and survival prediction. PLoS One. 2010;5(4):e10312.

16. Raponi M, Zhang Y, Yu J, et al. Gene expression signatures for predicting prognosis of squamous cell and adenocarcinomas of the lung. Cancer Res. 2006;66(15):7466-7472.

17. Davis S, Meltzer PS. GEOquery: a bridge between the Gene Expression Omnibus (GEO) and BioConductor. Bioinformatics. 2007;23(14):1846-1847.

**Supplementary figures**


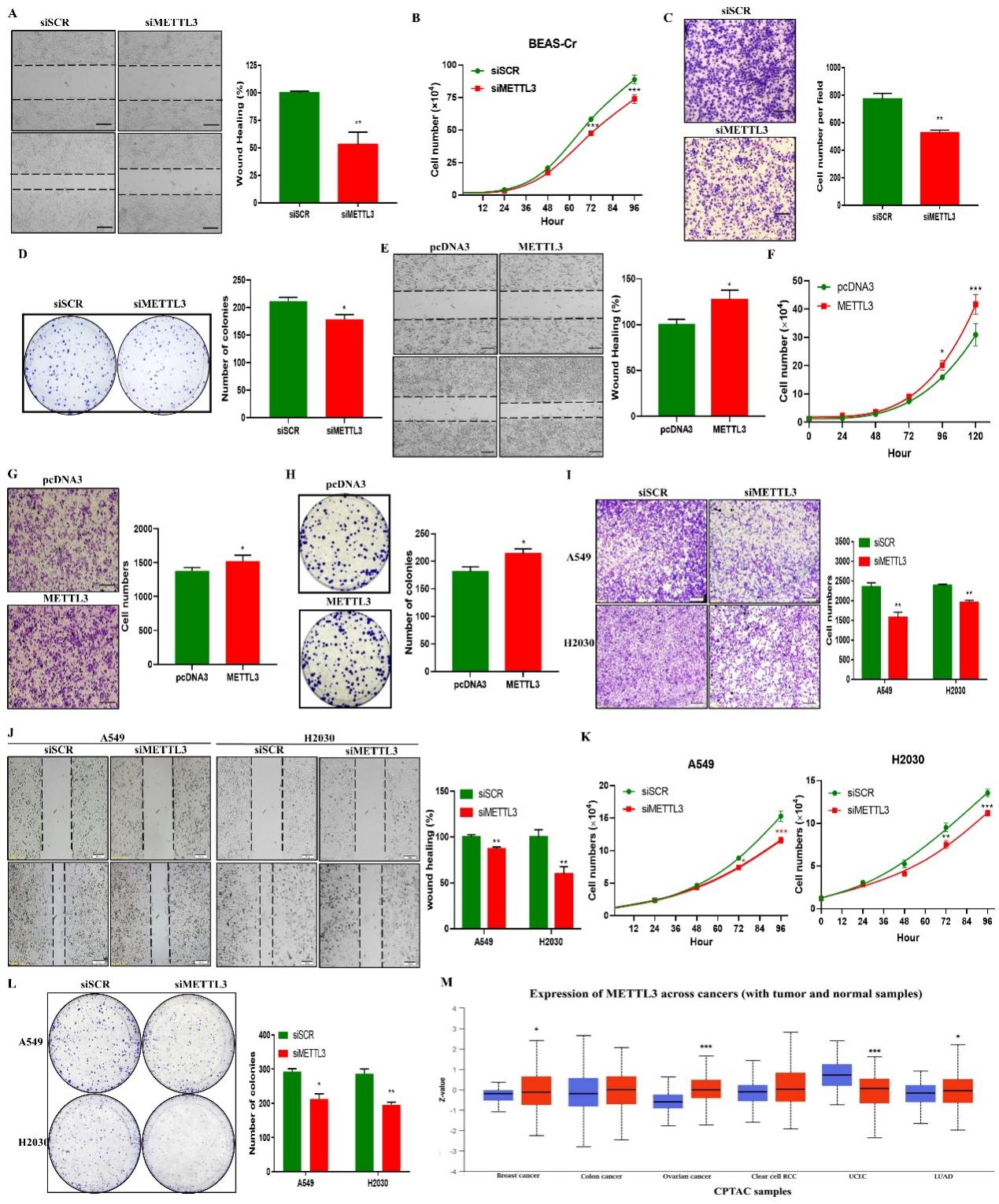


**Fig. S1: Identification of METTL3 roles in Cr (VI)-induced carcinogenesis and lung cancer development.** The wound-healing assay (A), cell proliferation assay (B), Transwell migration assay (C), and colony formation assay (D) were analyzed in the cells transfected with control siRNAs (siSCR) and METTL3 siRNAs (siMETTL3). The wound-healing assay (E), cell proliferation assay (F), Transwell migration assay (G), and colony formation assay (H) were analyzed in the cells transfected with an empty pcDNA3 vector and [pcDNA3/Flag-METTL3](https://www.addgene.org/53739/) plasmid. The Transwell migration assay (I), wound-healing assay (J), cell proliferation assay (K), and colony formation assay (L) were also analyzed in A549 and H2030 cells with control or METTL3 siRNAs (siSCR or siMETTL3). (M) METTL3 protein expression levels in different types of cancer by using UALCAN database. Scale bar refers to 100 μm. LUAD, lung adenocarcinoma. RCC, renal cell carcinoma. UCEC, Uterine corpus endometrial carcinoma. Data were presented as X+ SEM (n=3). *indicates significant difference between the groups at **P* < 0.05; **at *P* < 0.01; ***at *P* < 0.001.


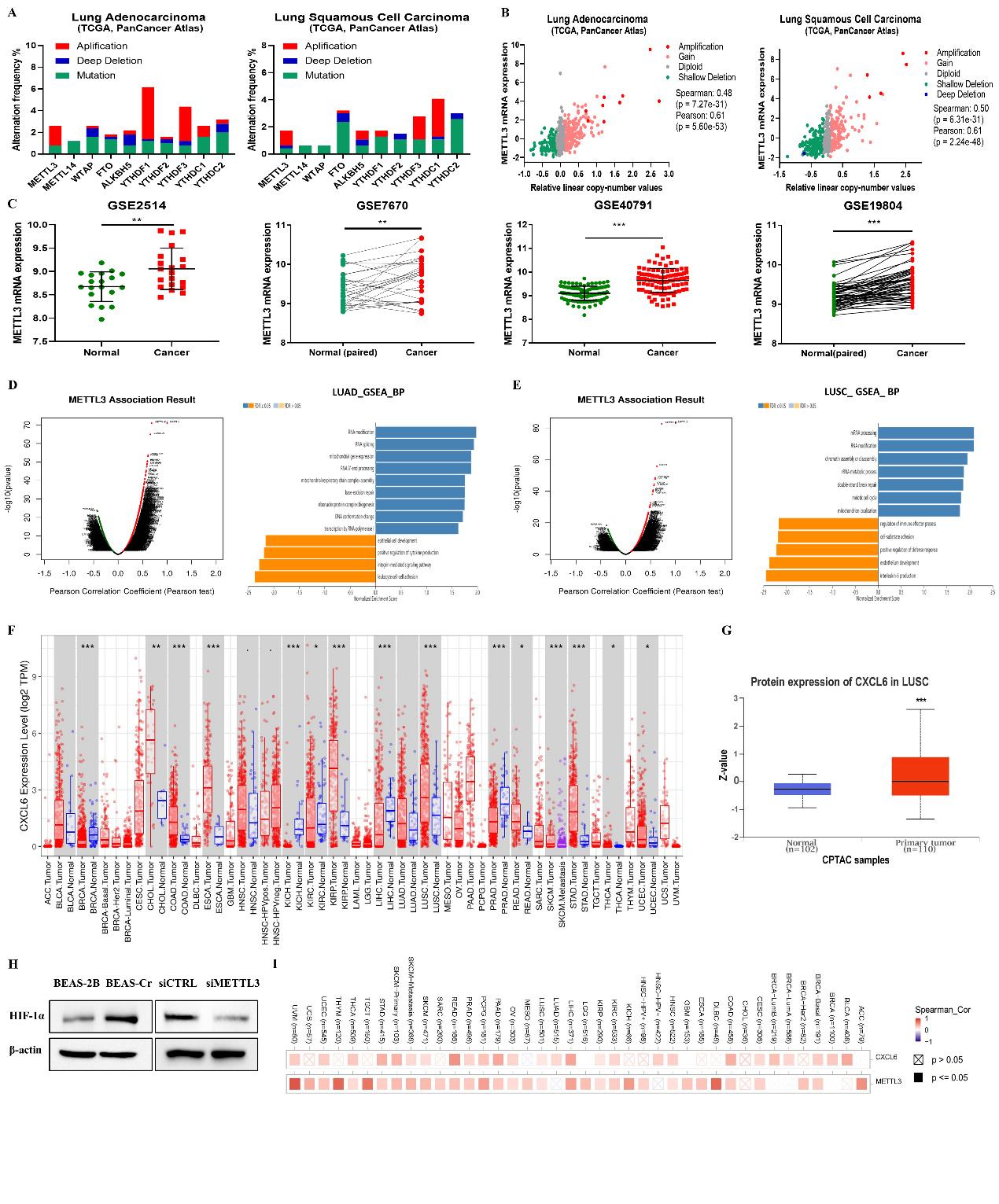


**Fig. S2: The expression levels, genetic alterations and potential function association of METTL3 in human lung cancer tissues.** (A) Genetic alterations of m6A-associated genes in TCGA-LUAD and TCGA-LUSC datasets. (B) Correlation analysis between gene expression levels and the copy number in TCGA lung cancer datasets. (C) METTL3 gene expression in lung tumors and normal counterparts is based on the results of four independent GEO datasets, including GSE2514, GSE7670, GSE40791, and GSE19804. The global gene levels associated with METTL3-high expression levels were identified by the Pearson test in TCGA-LUAD (D) and TCGA-LUSC (E) cohorts. Significantly enriched GO annotations of METTL3 in the LUAD cohort (D) and LUSC cohort (E). (F) CXCL6 mRNA expression levels were analyzed in lung tumor tissues and adjacent normal tissues across different types of cancer tissues. (G) CXCL6 protein expression levels in LUSC were analyzed by using UALCAN database. (H) Western blotting analysis of HIF-1α in BEAS-2B, Cr-T, and the cells expressing non-targeting control or METTL3 siRNAs (siSCR or siMETTL3). (I) The associations between HIF-1α and CXCL6, and HIF-1α and METTL3 levels were analyzed by using TIMER database. GO, Gene Ontology. TCGA, The Cancer Genome Atlas. LUAD, lung adenocarcinoma. LUSC, Lung squamous cell carcinoma. GEO, Gene Expression Omnibus. *indicates significant difference of groups at **P* < 0.05; ** at *P*< 0.01; *** at *P* < 0.001.
